# *Saksenaea vasiformis* Infection, French Guiana

**DOI:** 10.3201/eid1402.071079

**Published:** 2008-02

**Authors:** Denis Blanchet, Eric Dannaoui, Angela Fior, Florence Huber, Pierre Couppié, Nour Salhab, Damien Hoinard, Christine Aznar

**Affiliations:** * Centre Hospitalier Andrée Rosemon, Cayenne, French Guiana; † Institut Pasteur, Paris, France; ‡ Université Paris Descartes, Paris, France

**Keywords:** Zygomycota, French Guiana, antifungal agents, DNA sequence analysis, letter

**To the Editor:** The Zygomycetes are a class of filamentous fungi that are ubiquitous in the environment. Most of the species known to cause human or animal infections belong to a few genera within the order Mucorales. *Saksenaea vasiformis*, isolated from soil in India and described by Saksena in 1953, was reported to cause human infection for the first time by Ajello et al. (*1*). We report a case of a cutaneous lesion caused by *S. vasiformis* in French Guiana.

A nonimmunocompromised 47-year-old woman with a long history of non–type 1 diabetes mellitus, who had lived in French Guiana for many years, was admitted to Cayenne Hospital on November 18, 2005, with a cutaneous lesion of the abdominal wall and a fever that had lasted for 5 days before she was hospitalized. A skin biopsy specimen was obtained, and the first surgical debridement was performed on day 4 of hospitalization. A diagnosis of zygomycosis was made after direct examination and histopathologic examination of the tissue samples. Treatment was initiated on day 8, beginning with liposomal amphotericin B and itraconazole for 10 days, followed by liposomal amphotericin B alone for 12 days. Persistence of necrotic tissues at the infection site required additional surgical debridement on day 10. Histopathologic examination of the resected tissues showed damaged hyphae of zygomycetes. Resolution of clinical signs was excellent. Additional biopsy specimens taken by the end of treatment on day 21 were negative for fungi by direct examination and culture. Finally, a cicatrix was formed.

Histologic examination of the initial excised tissues showed a localized periumbilical cutaneous lesion of 14 cm × 13 cm. The skin was covered by a 1-mm layer of necrosis. The necrosis extended into all the abdominal adipose tissue at the rectus abdominis muscle and linea alba. Microscopy examination showed extensive superficial mycotic proliferation, with wide and irregular ribbonlike nonseptate hyphae and right-angle branching. These hyphae extended toward the hypodermic fat tissues and were associated with a break in the cell membrane of adipocytes and with crystals inside the adipocytes. These lesions were associated with massive nonsuppurative vascular thrombosis.

Culture of tissues samples on Sabouraud-chloramphenicol-gentamicin agar after 4 days at 30°C and 37°C grew a white aerial mold, which covered the entire surface of the agar. Examination by microscopy showed nonseptate sterile hyphae typical of a zygomycete. The fungal isolate was sent to the National Reference Center for Mycology and Antifungals at the Institut Pasteur, Paris. Subcultures on different media including malt extract agar and potato dextrose agar grew sterile mycelia. The isolates were then cultured in nutritionally deficient medium consisting of sterile distilled water supplemented with 0.05% filter-sterilized yeast extract (Difco, Becton, Dickinson and Company, Sparks, MD, USA) solution for 7 days at 37°C (*2*). Typical flask-shaped sporangia enabled identification of *S. vasiformis* (Figure). Sporulation also occurred on Czapek agar after 7 days’ incubation at 37°C.

**Figure:**
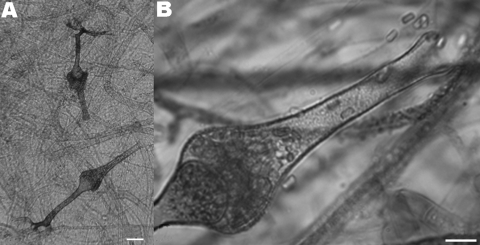
Microscopic characteristics of the isolate of *Saksenaea vasiformis* cultured on Czapek agar. A) Typical flask-shaped sporangia (scale bar = 25 μm) containing B) smooth-walled, rectangular sporangiospores (scale bar = 10 μm).

Molecular identification based on PCR amplification and sequencing of rDNA internal transcribed spacer (ITS) regions was also performed. Briefly, mycelia were grown in liquid Roswell Park Memorial Institute medium, and DNA was extracted as previously described (*3*). Ribosomal DNA, including the complete ITS1–5.8S-ITS2 region, was amplified with the fungal universal primer pairs V9D/LS266 (*4*) and ITS1/ITS4 (*5*), and both strands of PCR products were sequenced. The sequence has been deposited in GenBank (accession no. EU182902). Sequence alignment with the only *S. vasiformis* ITS sequence available in the GenBank database showed 82% similarity over 530 bp. This low degree of sequence homology is probably reflective of the need for further phylogenetic study of this genus.

Sporulation on Czapek agar enabled preparation of a sporangiospore suspension used for antifungal susceptibility testing. Sporangiospore suspension was counted microscopically and adjusted to the required density. MICs, determined by the EUCAST reference microdilution method (*6*), after 48 h of incubation were >8, 2, >8, 0.5, and >8 µg/mL for amphotericin B, itraconazole, voriconazole, posaconazole, and caspofungin, respectively. The MIC of 0.5 µg/mL for posaconazole suggests the potential clinical utility of this agent.

*S. vasiformis* has been isolated from soil samples in different parts of the world (*7*). This fungus has been rarely responsible for human infections. A recent review (*8*), which did not include infrequently cited articles (*9*,*10*), found only 30 human cases. This scarcity may occur because the diagnosis is often based on histologic features and *S. vasiformis* does not sporulate in routine mycology media.

Due to zygomycetes’ lack of susceptibility to most of the antifungal agents, identification of a zygomycete as the etiologic agent of an infection is essential for rapid and accurate management of the disease. Rare Zygomycetes species such as *S. vasiformis* or *Apophysomyces elegans* should be suspected when a nonsporulating zygomycete is isolated from an infected lesion. When this acute infection is suspected after examination of tissue by microscopy, the fungi should be cultured in specific culture media to induce sporulation or they should be identified by molecular tools.
